# Chloremia Disturbances in Critical Care: A Narrative Review of Pathophysiology, Clinical Impact and Management Strategies

**DOI:** 10.3390/life16010151

**Published:** 2026-01-16

**Authors:** Nicola Sinatra, Giuseppe Cuttone, Tarek Senussi Testa, Luigi La Via, Francesca Maria Rubulotta, Maurizio Giuseppe Abrignani, Carmelo Zumbino, Giuseppe Mulè, Giulio Geraci, Caterina Carollo

**Affiliations:** 1 Nephrology and Dialysis Unit, “Paolo Borsellino” Hospital, Azienda Sanitaria Provinciale Trapani, 91025 Marsala, Italy; sinatra.nicola@libero.it; 2 Anesthesia and Intensive Care, “Abele Ajello” Hospital, Azienda Sanitaria Provinciale Trapani, 91026 Mazara del Vallo, Italy; giuseppe.cuttone@hotmail.it; 3 Department of Cardiac Anesthesia and Intensive Care, Cardiovascular Network, IRCCS Policlinico San Martino Hospital, 16132 Genova, Italy; t.senussi@hotmail.it; 4 Department of Anesthesia and Intensive Care 1, University Hospital Policlinico “G. Rodolico–San Marco”, 95123 Catania, Italy; luigilavia7@gmail.com (L.L.V.); francesca.rubulotta@unict.it (F.M.R.); 5 Cardiology Unit, “Paolo Borsellino” Hospital, Azienda Sanitaria Provinciale Trapani, 91025 Marsala, Italy; maur.abri60@gmail.com; 6 Department of Emergency, “Paolo Giaccone” University Hospital, 90100 Palermo, Italy; carmelo.zumbino@policlinico.pa.it; 7 Department of Health Promotion Sciences, Maternal and Infant Care, Internal Medicine and Medical Specialties, “G. D’Alessandro” (PROMISE), University of Palermo, 90100 Palermo, Italy; giuseppe.mule@unipa.it; 8 Faculty of Medicine and Surgery, Kore University, 94100 Enna, Italy; giulio.geraci@unikore.it; 9 Unit of Nephrology and Hypertension, European Society of Hypertension Excellence Centre, University of Palermo, 90100 Palermo, Italy

**Keywords:** chloremia disturbances, electrolytes, critical care, fluid therapy, acute kidney Injury, sepsis

## Abstract

Chloride, the leading extracellular anion, plays a crucial role in acid-base balance, fluid homeostasis, and neuromuscular function. Despite historical underrecognition, emerging evidence demonstrates significant associations between chloremia disturbances and critical care outcomes. This paper aims to narratively review the pathophysiology, clinical features, and management strategies of chloremia disturbances in critically ill patients. Chloremia disturbances are common in ICU patients, with both hypochloremia (<96 mEq/L) and hyperchloremia (>106 mEq/L) independently associated with increased mortality, prolonged ICU length of stay, and organ dysfunction. In sepsis, chloride levels exhibit a prognostic value, with threshold effects around 105 mEq/L. Hyperchloremia particularly increases acute kidney injury risk, while hypochloremia correlates with prolonged mechanical ventilation. The choice of resuscitation fluids significantly influences clinical outcomes, with balanced crystalloids potentially reducing adverse events if compared to normal saline solutions. Recent large-scale trials demonstrate lower rates of major adverse kidney events with chloride-restrictive strategies. Optimal management requires careful patient monitoring along with acid-base assessment. Treatment approaches must identify underlying causes to avoid complications. Prevention strategies include protocol-based fluid therapy, medication selection consideration, and early intervention in high-risk patients. Emerging technologies, including continuous monitoring systems and machine learning algorithms, offer promising advances for predicting and managing chloride disturbances.

## 1. Introduction

Chloride, the most abundant anion in the extracellular fluid, plays a crucial role in multiple physiological processes including acid-base balance, fluid homeostasis, and neuromuscular function [1]. Despite its fundamental importance, chloride has historically been underestimated in clinical practice compared to other electrolytes such as sodium [2,3].

However, growing evidence over the past decade has underlined the significant impact of chloride disturbances on critical care outcomes [4,5], primarily through the recognition of metabolic acidosis associated with 0.9% saline administration [6,7], prompting a paradigm shift in fluid therapy and electrolyte management in Intensive Care Units (ICUs) [8,9].

Recent studies showed that chloride disturbances are common in critically ill patients, with hyperchloremia reported in 23–41% of septic and surgical ICU populations, and may be associated with adverse renal outcomes, particularly in patients with pre-existing kidney disease [10,11,12].

Dyschloremia is increasingly recognized as a prevalent electrolyte disorder: hypochloremia (Cl^−^ < 96–98 mmol/L) has been reported in up to 26.9% of acutely ill medical patients [13], with prevalence reaching 24% in patients hospitalized for acute worsening heart failure [14] and ranging from 6.7% to 35.1% in the ICU setting [15]. Hyperchloremia (Cl^−^ > 106–110 mmol/L) is even more prevalent in critical care, affecting 25–45% of ICU patients, with up to 75% developing transient hyperchloremia within the first 24 h of admission due to aggressive fluid resuscitation [16]. In septic shock, the incidence of hyperchloremia reaches 25.9% [17].

Both hypochloremia and hyperchloremia have been independently associated with adverse clinical outcomes, including increased mortality, prolonged ICU and hospital stay, and higher rates of organ dysfunction. Hypochloremia, in particular, has been shown to independently predict in-hospital mortality (OR 2.23; 95% CI: 1.29–3.86) and prolonged hospitalization [13]. The clinical relevance of chloride disturbances extends beyond their direct physiological effects to include alterations in hemodynamics, immune function, and renal perfusion [18,19,20].

These findings have led to the development of chloride-restrictive fluid protocols, although the optimal approach remains a subject of ongoing investigation [9,18,21,22,23,24].

This narrative review aims to provide a comprehensive and clinically oriented overview of chloride disturbances in critically ill patients. We will summarize the current understanding of chloride physiology and the pathophysiological mechanisms of dyschloremia. We will then analyze the clinical impact of both hypochloremia and hyperchloremia on patient outcomes across different critical care scenarios. Furthermore, we will examine the prognostic value of serum chloride as an independent biomarker of morbidity and mortality and critically evaluate the available evidence regarding fluid therapy strategies and their effects on chloride homeostasis. Finally, we will discuss practical management strategies and highlight emerging technologies for monitoring and treating chloride disturbances in the ICU setting.

## 2. Methods

We asked what is the current evidence regarding the clinical significance, prognostic value, and optimal management of chloride disturbances in critically ill patients, and how can this knowledge be translated into improved bedside care.

To address this question, a comprehensive literature search was conducted in PubMed/MEDLINE, Scopus, Web of Science, and the Cochrane Library databases from inception through December 2025. The search strategy employed a combination of Medical Subject Headings (MeSH) terms and free-text keywords, including: (“chloride” OR “chloremia” OR “hypochloremia” OR “hyperchloremia” OR “dyschloremia” OR “serum chloride”) AND (“critically ill” OR “intensive care” OR “ICU” OR “critical care” OR “sepsis” OR “septic shock” OR “acute kidney injury” OR “AKI” OR “heart failure” OR “fluid therapy” OR “crystalloid” OR “saline” OR “balanced solution” OR “mortality” OR “outcomes” OR “prognosis”).

Additional relevant articles were identified through manual screening of reference lists from key publications, recent systematic reviews, and meta-analyses. We also searched clinical trial registries (ClinicalTrials.gov) to identify ongoing relevant studies.

Inclusion criteria comprised: (a) original research articles (observational studies, randomized controlled trials); (b) systematic reviews and meta-analyses; (c) authoritative narrative reviews providing mechanistic insights; and (d) clinical practice guidelines. Articles published in English were prioritized, although seminal non-English publications with available translations were considered. Case reports, editorials, and conference abstracts were excluded unless they provided unique pathophysiological insights not available elsewhere.

Given the narrative nature of this review, no formal quality assessment or risk of bias evaluation of individual studies was performed. However, in synthesizing the evidence, preference was given to large-scale prospective cohort studies and randomized controlled trials, recent systematic reviews and meta-analyses, studies with appropriate adjustment for confounding variables, and publications in peer-reviewed journals with established impact. Where conflicting evidence existed, we aimed to present multiple perspectives and acknowledge areas of ongoing uncertainty.

## 3. Physiology of Chloride Homeostasis

The plasma Chloride concentration is between 96 and 106 mEq/L [1,2]. In adults the total body Chloride content is 115–120 mEq/kg, with approximately 85% located in the extracellular space [25], while an intracellular concentration is actively maintained at lower levels (4–30 mEq/L) through energy-dependent transport mechanisms [25]. This distribution is crucial for neurons and muscle cells where Chloride gradients influence membrane potential and cellular excitability [26]. Chloride homeostasis is maintained through complex regulatory mechanisms involving multiple organs. The kidneys play a pivotal role, with approximately 180 g of Chloride filtered daily and 99% reabsorbed under normal conditions [27].

Renal Chloride handling involves several transporters, including the Na-K-2Cl cotransporter (NKCC2) in the thick ascending limb and the pendrin-CFTR system in the collecting duct [28]. These mechanisms are regulated by various hormones, including aldosterone and angiotensin II [29]. Chloride plays a crucial role in the strong ion difference (SID), that on its turn is a key determinant of acid-base status according to the Stewart approach [30,31]. Changes in plasma Chloride concentration directly affect the SID, leading to metabolic acid-base disturbances independently of bicarbonate changes [32]. Chloride also interacts with other electrolytes, particularly sodium and potassium. The maintenance of electroneutrality requires that changes in sodium concentration be accompanied by proportional changes in anions, primarily Chloride [33]. This relationship becomes particularly important in critical illness, where fluid and electrolyte shifts can rapidly alter Chloride homeostasis [34].

## 4. Measurement and Assessment

The accurate measurement and interpretation of Chloride levels is essential for decision-making. Although multiple methods are available for Chloride assessment [35], the gold standard for Chloride measurement remains ion-selective electrode (ISE) technology, both direct and indirect ISE [36]. Direct ISE, used in point-of-care testing, measures Chloride activity in undiluted samples, while indirect ISE, more frequent in centralized laboratories, requiring sample dilution [37]. From this distinction arise significant clinical implications. Indirect ISE methods assume a normal plasma water fraction (approximately 93% of plasma volume), but this assumption becomes invalid in patients with severe hyperlipidemia or hyperproteinemia. In such conditions, the solid phase of plasma (lipids and proteins) occupies a greater proportion of the sample volume, reducing the aqueous phase where electrolytes are dissolved. Upon dilution, the measured chloride concentration is erroneously calculated based on total plasma volume rather than plasma water, leading to pseudohypochloremia—falsely low chloride readings despite normal chloride activity in plasma water [37,38].

The clinical consequences of unrecognized pseudohypochloremia can be substantial. Clinicians may initiate unnecessary chloride supplementation with normal saline or hypertonic saline solutions, potentially leading to iatrogenic fluid overload, true hyperchloremia, and hyperchloremic metabolic acidosis [18,39]. Furthermore, pseudohypochloremia can significantly affect acid-base interpretation: the calculated anion gap may appear falsely elevated, mimicking high anion gap metabolic acidosis and prompting unnecessary diagnostic workups for lactic acidosis, ketoacidosis, or toxic ingestions [30,31]. The strong ion difference (SID) calculation is similarly affected, potentially leading to misclassification of acid-base disorders and inappropriate therapeutic interventions [32]. In critically ill patients where precise acid-base management is essential, such errors can delay recognition of the true underlying disorder and adversely impact clinical outcomes [5].

The clinical impact of these measurement artifacts is particularly relevant in ICU populations, where hypertriglyceridemia (from propofol infusions or parenteral nutrition), paraproteinemias, and hypoalbuminemia are common. Clinicians should maintain a high index of suspicion for pseudohypochloremia when encountering unexplained hypochloremia in patients with known lipid or protein abnormalities, or when the measured chloride value is discordant with the overall clinical and acid-base picture [35,37]. Practical strategies to identify and address this artifact include requesting direct ISE measurement (typically available through point-of-care blood gas analyzers), comparing central laboratory values with point-of-care results, calculating the sodium-chloride difference to assess for unexpected discrepancies, and reviewing the lipid panel and protein levels [40,41]. When pseudohypochloremia is suspected, treatment decisions should be based on direct ISE measurements or deferred until the true chloride status is clarified, thereby avoiding potentially harmful interventions [5,42].

Blood gas analyzers typically use direct ISE technology and are prevalent in ICU [26,27], but they need a careful quality check and regular calibration [42]. Changes in plasma water content, protein and lipid abnormalities, concurrent acid-base disturbances, impact of various therapeutic interventions complicate the interpretation of chloride measurements in critically ill patient [5].

While traditional reference ranges (96–106 mEq/L) are widely accepted [21], optimal chloride levels may vary depending on the clinical context [21], particularly during sepsis or acute kidney injury, where Chloride homeostasis may be significantly altered [22]. Age, sex, and ethnicity-specific variations should also be considered when interpreting results [9].

## 5. Hypochloremia

Hypochloremia, defined as serum chloride concentration below 96 mEq/L, is a common electrolyte disturbance that has been increasingly recognized as an independent predictor of poor outcomes [5]. Table 1 shows classification and underlying mechanisms (Chloride loss versus dilution) of hypochloremia and patterns of associated electrolyte disturbances [1,2,43]. The activation of the renin–angiotensin–aldosterone system, effects of inflammations, alterations in renal Chloride handling and the impact of mechanical ventilation on acid-base balance could lead to the development of hypochloremia in critical illness [33,44].

In ICU, hypochloremia frequently results from diuretic therapy, particularly loop diuretics, which significantly affect chloride homeostasis [45]. Other common causes include ongoing losses through vomiting or nasogastric drainage, third-space fluid accumulation, administration of large volumes of hypotonic fluids, respiratory alkalosis and post-hypercapnia syndrome [46]. The clinical presentation of hypochloremia includes metabolic alkalosis, neuromuscular symptoms, cardiovascular effects, respiratory compensation, and altered mental status [47]. These manifestations can significantly differ among patients and may be masked or complicated by underlying critical illness.

Significant associations between hypochloremia and adverse clinical outcomes are known [48]. Patients with hypochloremia experience increased mortality rates, prolonged mechanical ventilation requirements, extended ICU stays, higher rates of acute kidney injury, and increased healthcare costs [49]. These associations persist even after adjusting for illness severity and other confounding factors, suggesting an independent role of hypochloremia in determining patient outcomes.

## 6. Hyperchloremia

Hyperchloremia, characterized by serum chloride levels exceeding 106 mEq/L, is a frequent electrolyte disturbance in ICU setting. Recent evidence from a systematic review and meta-analysis indicates a pooled prevalence of 34% (95% CI: 26–43%) in critically ill adults, with reported rates varying widely (9–79%) across different ICU populations [16]. This condition is increasingly recognized for its independent impact on patient outcomes, particularly in the context of fluid resuscitation, where it has been associated with increased mortality and acute kidney injury [2,4].

Hyperchloremia can be categorized based on severity and underlying mechanisms (Table 2) [50]. The classification also distinguishes hyperchloremic metabolic acidosis from other acid-base disturbances [32].

The pathophysiological mechanisms of hyperchloremia in critical illness include an overload of Chloride, particularly through unbalanced crystalloids, particularly 0.9% saline [39]. Other mechanisms are impaired renal chloride excretion, tissue hypoperfusion, diabetes insipidus, drugs (several antibiotics and total parenteral nutrition), endocrine disorders and alterations in strong ion difference [51]. The resulting acid-base disturbances can lead to significant cellular dysfunction and organ injury [18,52]. The recognition of iatrogenic causes has led to significant changes in fluid management strategies in critical care [53].

The clinical presentation of hyperchloremia includes metabolic acidosis, reduced renal blood flow, acute kidney injury, and various manifestations of organ dysfunction [23]. Cardiovascular effects (such as reduced myocardial contractility) and neurological manifestations (from mild confusion to severe encephalopathy [10] are the leading clinical features.

Hyperchloremia is related with adverse outcomes in ICU, such as increased mortality, longer stays, higher rates of acute kidney injury and increased need of renal replacement therapy [11] especially in patients with sepsis or undergoing major surgery [54]. In a retrospective study on adult patients admitted to the ICU, increased chloride levels 48 h after admission were associated with increased incidence of major adverse kidney events within 30 days [55]. In another prospective study, mortality rate was higher (60% vs. 46%) and survival time was lower (19.0 ± 1.46 vs. 23.0 ± 4.36 days; *p* = 0.035) in patients with high Cl levels compared to the patient group with normal or low Cl levels. In the Cox regression analysis, the survival time of the patients was associated with Cl (hazard ratio: 1.030 (1.008–1.049), *p* < 0.05) [56].

## 7. Special Clinical Scenarios

The impact of Chloremia disturbances varies significantly across different critical care scenarios. Understanding these specific contexts is crucial for optimizing patient care and outcomes [57].

### 7.1. Sepsis

Sepsis continues to be a threatening medical issue. In this condition, inflammatory mediators and hemodynamic alterations significantly impact electrolyte balance [58]. Serum chloride has emerged as a potential prognostic marker in sepsis. Both hyper- and hypochloremia in septic patients are associated with increased mortality and organ dysfunction [4]. Data from the Medical Information Mart for Intensive Care IV (MIMIC-IV) about 3726 septic shock patients, showed that hypochloremia was significantly associated with increased mortality and increased incidence of AKI after adjusting for several variables [51]. Another retrospective cohort study from the MIMIC-IV database on a total of 17,743 patients with sepsis demonstrated an L-shaped association between serum chloride levels and 365-day mortality in sepsis patients, with higher serum chloride levels corresponding to a lower mortality risk [59]. A third study from the MIMIC IV database incorporated 6219 adult sepsis cases. Four distinct serum chloride trajectories were identified: Patients in Class 4, characterized by elevated serum chloride levels, had the highest mortality both in 28 days (HR:2.04, 95% CI 1.53–2.71) and 365 days (HR: 1.90, 95% CI 1.52–2.37) after adjusting for confounding factors [60].

### 7.2. Renal Diseases

In ICU setting, acute kidney injury (AKI) is one of the most common complications, affecting approximately 30–60% of critically ill patients and representing a major contributor to morbidity and mortality [16,61]. The relationship between Chloremia disturbances and acute kidney injury (AKI) is bidirectional. Hyperchloremia is an independent risk factor for AKI development, particularly in patients receiving large volumes of chloride-rich fluids [9]. On the contrary, AKI itself can lead to chloride abnormalities through impaired excretion and altered acid-base homeostasis [62]. In a single-center, retrospective study in patients receiving hemodialysis, the cumulative daily intake of chloride was independently associated with one-year mortality in separate multivariable cox proportional hazards models [63]. In another longitudinal observational study on 273 patients, serum chloride levels were associated with AKI [64]. High chloride levels were identified as risk factors for increased mortality also in acute tubular necrosis patients [65].

### 7.3. Surgical and Traumatic Patients

Perioperative Chloremia disturbances significantly impact postoperative outcomes [52]. Large-volume fluid administration during major surgery can lead to hyperchloremic metabolic acidosis, while postoperative fluid shifts and losses may result in hypochloremia [39]. Balanced solutions show potentially better outcomes compared to chloride-rich alternatives [53]. Trauma patients present unique challenges in Chloride management due to massive hemorrhage, fluid resuscitation, and tissue injury [66]. The initial resuscitation phase often involves large-volume fluid administration, potentially leading to significant Chloride load. Recent evidences suggest that balanced resuscitation strategies may improve outcomes in trauma patients [67]. In a study from the MIMIC-IV database, focused on 10,996 individuals admitted to the surgical/trauma Surgical ICU, the multivariable Cox regression suggested a substantial inverse association between high serum chloride levels and decreased mortality at 30 days (hazard ratio [HR]: 0.96; 95% CI: 0.95–0.97; *p* < 0.001), 90 days (HR: 0.97; 95% CI: 0.96–0.98; *p* < 0.001), and 180 days (HR: 0.97; 95% CI: 0.96–0.98; *p* < 0.001). Particularly, patients in the highest quartile of serum chloride faced significantly lower mortality risks compared to those in the lowest quartile (30 days HR = 0.65, 90 days HR = 0.71, 180 days HR = 0.69, *p* < 0.001). RCS analysis depicted an L-shaped curve. From a concentration of 104 mEq/L, a decrease in serum chloride levels was associated with an increased risk of mortality [68].

### 7.4. Cardiovascular Diseases

Electrolyte imbalances are commonly observed in myocardial infarction (MI) [69]. Additionally, a correlation between serum sodium, whose levels have been linked to unfavorable outcomes, and serum chloride has been observed. A retrospective cohort study analysis on the Na/Cl ratio exhibited an independent association between this index and in-hospital mortality (HR = 1.28; 95% CI: 1.11–1.47, *p* < 0.001). A further analysis showed a nonlinear relationship between the same index and in-hospital mortality among patients with MI, with a threshold at approximately 1.37 [70].

Patients with heart failure experience complex interactions between chloride homeostasis and cardiovascular function [71]. Diuretic therapy, a cornerstone of heart failure management, frequently leads to chloride depletion. Furthermore, the neurohormonal activation in heart failure significantly impacts chloride handling, often complicating management strategies [72].

Three retrospective studies investigated the role of chloride in heart failure patients from the MIMIC IV database. In 15,983 participants admitted in ICU due to congestive heart failure, the groups with the highest and lowest blood chloride levels exhibited increased in-hospital mortality, with fully adjusted ORs of 1.36 [95% CI: 1.08–1.71] and 1.25 (95% CI: 1–1.56), respectively. A U-shaped relationship was observed between blood chloride levels and in-hospital mortality, with the lowest risk observed at a threshold of 105.017 mEq/L [73]. In a second study, a total of 7844 patients with acute heart failure were divided into three groups based on the Na/Cl ratio level upon admission. The multivariable Cox regression analysis revealed that the baseline Na/Cl ratio significantly elevated the risk of in-hospital mortality among critically ill patients (HR = 1.34, 95% CI: 1.21–1.49). Furthermore, when the Na/Cl ratio was converted into a categorical factor and the initial tertile was taken as a point of comparison, the HRs and 95% (CIs) for the second and third tertiles were 1.27 (1.05–1.54) and 1.53 (1.27–1.84), respectively. ROC curve analysis showed that Na/Cl ratio had a more sensitive prognostic value in predicting in-hospital mortality of AHF than the sodium or chloride level alone (0.564 vs. 0.505, 0.544). Subgroup examinations indicated that the association between the Na/Cl ratio upon admission and the mortality rate of critically ill patients with AHF remained consistent in the subgroups of hyponatremia and hypochlorhydria (*p* for interaction > 0.05). The linear relationship between the Na/Cl ratio and in-hospital mortality in AHF patients indicates a positive association [74]. In a third study on 7063 adult patients with coexisting diabetes and congestive heart failure, an inverse association between serum chloride levels and in-hospital mortality has been found. The relationship between serum chloride and in-hospital mortality demonstrated a linear behavior (non-linear *p* = 0.958) [75]. In another study, a total of 9364 HF patients hospitalized in the ICU were enrolled. Patients were divided into hypochloremia (<96 mEq/L), normal chloride (96–108 mEq/L), and hyperchloremia (>108 mEq/L) groups. Hypochloremia was associated with a higher risk of in-hospital mortality than normal chloride (OR 1.54, 95% CI 1.26–1.86, *p* < 0.001), hyperchloremia was not significantly related to in-hospital mortality (OR 1.00, 95% CI 0.85–1.19, *p* = 0.962). However, a linear association between serum chloride and in-hospital mortality was found in the low and normal bicarbonate groups (all *p* for nonlinear > 0.05) [76].

### 7.5. Pulmonary Diseases

Chloride also plays a crucial role in acid-base homeostasis during respiratory failure [77]. Mechanical ventilation and associated treatments can significantly impact chloride balance, while severe respiratory acidosis or alkalosis can lead to compensatory chloride shifts. The management of Chloride abnormalities in these patients requires careful consideration of the underlying respiratory disease [78]. Data from the MIMIC-IV database were extracted for analysis in critically ill patients with chronic obstructive pulmonary disease. Higher quartiles of serum chloride levels were associated with significantly lower levels of weight, red blood cells, platelet, and hemoglobin (*p* < 0.05), accompanied by lower 90-day and 365-day mortality (*p* < 0.05). Cox proportional hazard model indicated that the risk of death was significantly lower in the fourth quartile of serum chloride levels compared with the first quartile after adjusting for confounders (90-day HR = 0.54, 365-day HR = 0.52, both *p* < 0.05). An L-shape relationship was observed, with risks of death decreasing as serum chloride levels increased, although the magnitude decreased when levels reached 102 mmol/L [79].

### 7.6. Cerebrovascular Diseases

In a retrospective study of 376 patients diagnosed with intracerebral hemorrhage and included in the MIMIC-III from 2001 to 2012, an increase in chloride (>5 mmol/L) was associated with a higher OR for 90-day mortality [80].

### 7.7. Coronavirus Disease—2019 Disease (COVID-19)

Serum chloride levels are recognized as a prognostic biomarker in critically ill COVID-19 patients. In a total of 390 patients with COVID-19, serum chloride levels upon admission were markedly lower in tocilizumab users, patients requiring ICU care, and patients who died [81]. In 265 adult COVID-19 patients admitted to an ICU between October 2020 and March 2021, the standard drug solvent sodium chloride 0.9% was employed in 161 patients and glucose 5% in 104. Severe hypernatremia occurred less frequently in the glucose 5% group (6.6% vs. 20%) [82].

### 7.8. Pediatric Population

A retrospective cohort study was conducted by screening pediatric patients diagnosed with metabolic acidosis from a pediatric ICU database. The risk of in hospital death decreased when chloride was ≥113 mEq/L (adjusted OR = 0.365, 95% CI: 0.217–0.614, *p* < 0.001). Sodium bicarbonate treatment can reduce the odds of mortality in pediatric patients with hyperchloremia, regardless of their lactate levels [83] (Table 3).

## 8. Fluid Therapy and Chloride

The relationship between fluid therapy and Chloride homeostasis has become increasingly recognized as a crucial aspect of critical care medicine. The choice of resuscitation fluids significantly influences chloride balance and subsequent clinical outcomes [7]. Fluid administration practices in ICU changed in recent years with a trend toward more restrictive fluid strategies [34,84]. These changes could have influenced the electrolyte disturbances in ICU patients. Traditional 0.9% saline contains a supraphysiologic chloride concentration (154 mEq/L) compared to plasma (96–106 mEq/L), while balanced solutions contain chloride concentrations closer to physiological levels [18].

Large-scale randomized trials showed that balanced crystalloids may reduce the incidence of adverse outcomes, including acute kidney injury and mortality, compared to 0.9% saline [21]. The SMART trial showed significantly lower rates of major adverse kidney events when balanced crystalloids were used instead of saline solutions in critically ill adults [85]. In a randomized controlled trial, 4823 ICU patients receiving intravenous fluid therapy with balanced solution versus 0.9% sodium chloride (saline) were divided into cohorts according to quartiles of baseline serum chloride concentration. The risk-adjusted OR (95% CI) for day-90 mortality for patients assigned balanced solution compared to saline was 1.23 (0.95–1.58), 0.95 (0.73–1.25), 0.88 (0.64–1.21), and 0.76 (0.57–1.01) for lowest to highest chloride subgroups, respectively (*p* value for interaction = 0.10). No significant differences were found between balanced compared to saline solutions according to baseline serum chloride concentrations [86].

Volume and timing of fluid administration significantly influence Chloride homeostasis. Rapid administration of large volumes of saline could cause renal vasoconstriction and reduced glomerular filtration rate: such effects were not observed with balanced solutions [87].

A Chloride-restrictive strategy has been associated with decreased incidence of acute kidney injury and need for renal replacement therapy [9]. However, the optimal threshold for Chloride restriction remains debated [53].

The choice of resuscitation fluid should take in account multiple factors, including the underlying disease, the acid-base status, and organ function [8,34]. The timing of transition from initial resuscitation to maintenance fluids also impacts chloride homeostasis and should be carefully evaluated [88].

Continuous monitoring of Chloride levels and acid-base status during fluid therapy is essential. Dynamic assessment of fluid responsiveness should guide fluid therapy decisions [89]. The development of protocols incorporating both Chloride monitoring and fluid management has shown promising in improving patient outcomes [90].

In diabetic ketoacidosis (DKA), fluids with high Na content are preferred due to the association of Na with β-Hydroxybutyrate (β-HB) and renal excretion of Na-β-HB. However, these fluids may cause hyperchloremic metabolic acidosis due to their high chloride concentration.

Base-excess chloride (BECl) has been suggested as a better approach for assessing the effect of chloride on acid–base status. In a retrospective study including 35 DKA patients in tertiary ICUs, subjects were divided into two groups according to the Na-Cl difference of the administered fluids during the first 6 h of treatment: Group I [GI, fluids with Na-Cl difference = 0, chloride-rich group] and Group II [GII, fluids with Na-Cl difference > 32 mmol, chloride non-rich group]. The main fluid administered in GI was 0.9% NaCl, whereas in the GII, it was bicarbonate, Isolyte-S, and 0.9% NaCl. In GI, the chloride load administered was higher; the BECl level of the fluids was lower than in GII. After 6 h, although sodium and strong ion gap values were similar, patients in GI were more acidotic due to iatrogenic hyperchloremia and, as a result, were more hypocapnic than GII. Thus, administering chloride-rich fluids may help reduce unmeasured anion acidosis in DKA [91].

Some ongoing studies are focused on fluid administering in the ICUs.

- The FLUID-ICU study (“Fluid administration and fluid accumulation in intensive care units—an international inception cohort study”) is a prospective international 14-day inception cohort study with a minimum sample size of 1000 patients from more than 50 ICUs. The primary outcomes of this sub-study are the proportion of patients with hyperchloremia, and hypochloremia. The study will assess days alive without the use of life support at Day 90, and risk factors for developing disturbances in chloride including disease severity by SMS-ICU score, type of ICU, use of diuretics, and presence of fluid accumulation. Furthermore, days alive and out of hospital and mortality at Day 90 will be descripted [92].
- The ‘Balanced Electrolyte Solution versus Saline Trial for Diabetic Ketoacidosis’ (BEST-DKA) trial (from March 2024) is a Phase 3 cluster-crossover, blinded, pragmatic, randomized, controlled trial comparing the effects of 0.9% saline or buffered crystalloid solution in patients with moderate to severe DKA treated in emergency department and/or ICUs to detect a 2-day increase in the primary outcome, days alive and out of hospital to day 28 [93].
- The multicentre, open-label, “Sweet-water” trial will include a projected total sample size of 4485 patients, to test the effectiveness of glucose 5% solution as the default drug diluent instead of sodium chloride 0.9%. The primary endpoint is the prevalence of hypernatraemia > 150 mmol/L through day 28. The number of days alive and free of the ICU through day 28 will be tested hierarchically as a key secondary endpoint. Other exploratory endpoints include ICU mortality, ICU-free days, hospital-free days and other clinical outcomes [94].

While glucose 5% as a drug diluent represents a promising strategy to reduce iatrogenic sodium and chloride load, clinicians should be aware of potential risks associated with its use. First, the administration of electrolyte-free solutions may predispose patients to hyponatremia, particularly in those with impaired free water excretion due to syndrome of inappropriate antidiuretic hormone secretion (SIADH), heart failure, liver cirrhosis, or renal dysfunction. In critically ill patients, non-osmotic stimuli for antidiuretic hormone release (pain, nausea, stress, mechanical ventilation) are common, further increasing susceptibility to hyponatremia [95]. Second, glucose-containing solutions may contribute to hyperglycemia, which is independently associated with adverse outcomes in critically ill patients, including increased infection rates, impaired wound healing, and mortality [96]. This risk is particularly relevant in patients with diabetes mellitus, stress-induced hyperglycemia, or those receiving corticosteroids or parenteral nutrition [97]. Third, rapid glucose metabolism results in the generation of free water, which may exacerbate hyponatremia if not appropriately monitored [98]. Therefore, implementation of glucose 5% as a default drug diluent requires careful patient selection, frequent monitoring of serum sodium and glucose levels, and individualized adjustments based on the patient’s metabolic and fluid status. The ongoing Sweet-water trial will provide important data on the safety profile of this approach and help define optimal patient populations for this strategy

## 9. Monitoring and Management

Continuous monitoring of Chloride levels must be integrated with clinical and laboratory parameters. The frequency of monitoring should be tailored to illness severity and the rate of clinical change [40,99].

Management of hypochloremia requires careful attention both to the underlying cause and the rate of correction. Replacement strategies must consider the total chloride deficit, concurrent electrolyte abnormalities, and acid-base status [44]. The choice of replacement fluid should be guided by the specific clinical context, with options ranging from normal saline to custom-made electrolyte solutions [18]. Addressing the underlying cause, such as discontinuing diuretics or managing gastrointestinal losses, is crucial for successful treatment [43].

Correction of hyperchloremia focuses on reducing Chloride intake and promoting appropriate excretion. This may involve switching to balanced crystalloids, adjusting medication regimens, and ensuring adequate renal function [9]. The rate of correction should be controlled to prevent rapid shifts in osmolality and associated complications [95]. In cases of severe hyperchloremia, renal replacement therapy may be necessary, particularly when complicated by acute kidney injury or severe metabolic acidosis [100].

**Figure 1:**
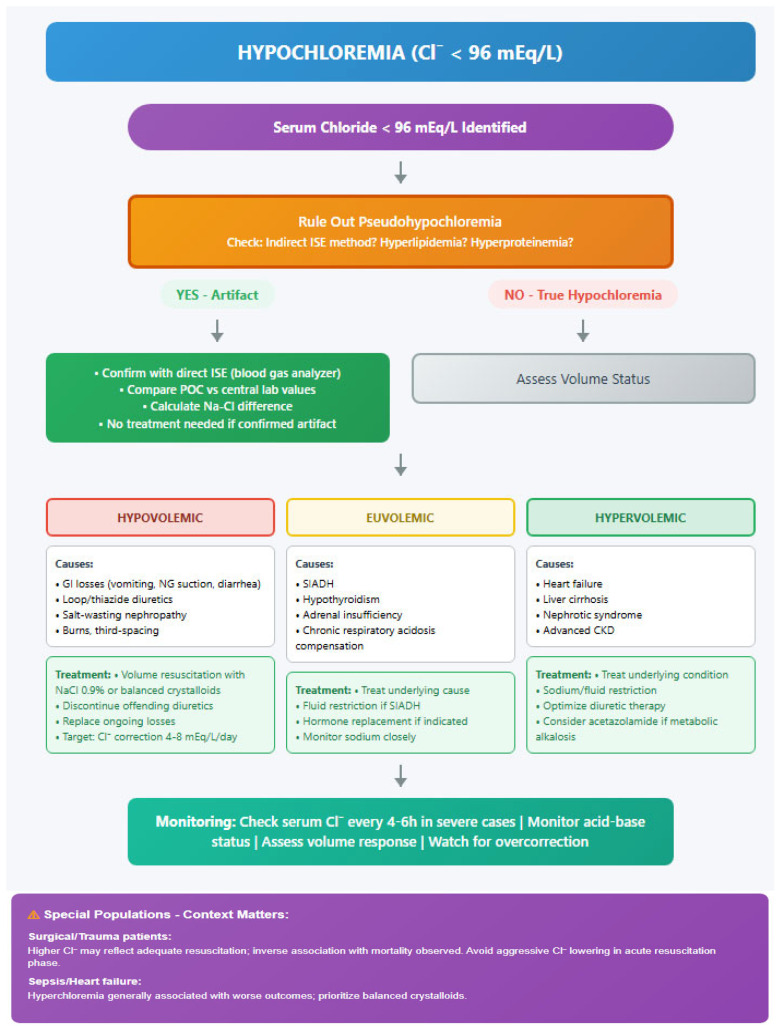

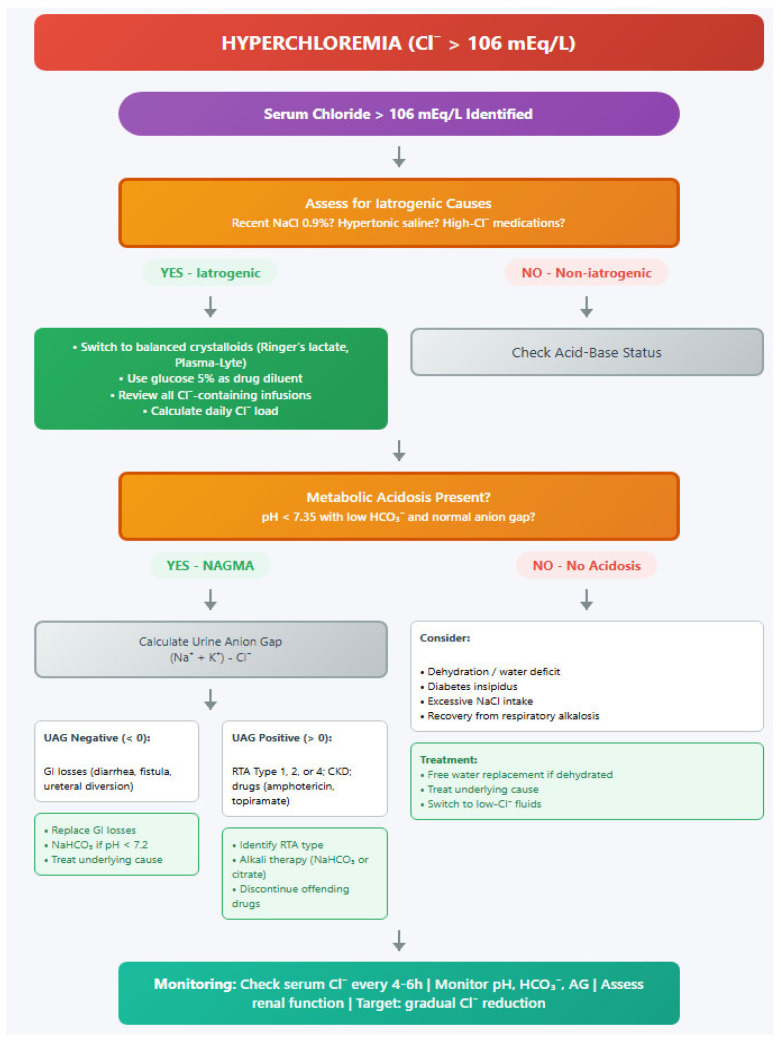
**Management Algorithm for Chloride Disturbances in Critically Ill Patients**. ISE = Ion-Selective Electrode; POC = Point-of-Care; NG = Nasogastric; SIADH = Syndrome of Inappropriate Antidiuretic Hormone; CKD = Chronic Kidney Disease; NAGMA = Normal Anion Gap Metabolic Acidosis; UAG = Urine Anion Gap; RTA = Renal Tubular Acidosis; AG = Anion Gap.

Prevention of Chloremia disturbances involves careful attention to fluid management, medication selection, and early recognition of risk factors [19]. Implementation of protocols for fluid therapy, regular monitoring of electrolyte status, and early intervention in high-risk patients can help prevent significant chloride abnormalities [22,34,100]. The rate of correction should be guided by the severity of the disturbance, underlying pathology, and presence of symptoms [8]. Continuous monitoring during correction is essential to prevent overcorrection and associated complications (Figure 1).

## 10. Future Directions

The field of Chloride homeostasis in ICU continues to evolve, with emerging research and technological advances offering new perspectives and opportunities for improved patient care. Understanding these developments is crucial for advancing clinical practice and research initiatives [50].

The optimal targets for Chloride levels in different critical care scenarios remain undefined, and the impact of Chloride variability on outcomes needs further exploration [4,18,101].

Technological advances are revolutionizing the monitoring of Chloride homeostasis. Continuous electrolyte monitoring systems are under development, promising real-time assessment of Chloride levels and other electrolytes [102]. Machine learning algorithms are being developed to predict Chloride disturbances and their clinical consequences, potentially allowing for earlier intervention [103]. Integration of these technologies with electronic health records may enable more sophisticated clinical decision support systems [61].

Innovation in therapeutic interventions continues to expand treatment options. Development of new balanced crystalloid solutions with optimized electrolyte composition shows promise for preventing Chloride-related complications [104]. Novel drug delivery systems and therapeutic agents targeting specific aspects of Chloride transport are under investigation [34]. The potential for personalized fluid therapy based on genetic and molecular markers represents an exciting frontier in ICU [105].

Implementation science focuses on translating research findings into clinical practice. Development of standardized protocols for Chloride monitoring and management, incorporating latest evidence, may improve care consistency [23]. Quality metrics specific to Chloride management are being developed to assess and improve care delivery [106]. Integration of these initiatives with existing critical care bundles may enhance overall patient outcomes [107].

### Implications for Clinical Practice

The accumulating evidence on chloride dysregulation in critically ill patients has important implications for bedside practice. Clinicians should consider integrating the following principles into their daily management of ICU patients.

**Routine Chloride Monitoring.** Serum chloride should be monitored regularly in all critically ill patients, not merely as part of a basic metabolic panel but as a distinct parameter warranting clinical attention. Given that both hyperchloremia and hypochloremia are independently associated with adverse outcomes, establishing baseline values on ICU admission and tracking trends over time is essential [4,101]. Particular vigilance is warranted in patients receiving large-volume fluid resuscitation, those with sepsis or acute kidney injury, and patients on continuous renal replacement therapy.

**Evidence-Based Fluid Selection.** The choice of intravenous fluids should be guided by chloride content and the patient’s current chloride status. In patients with normal or elevated serum chloride, balanced crystalloid solutions (such as Ringer’s lactate or Plasma-Lyte) are preferable to 0.9% saline, which contains supraphysiological chloride concentrations (154 mEq/L) [18,23,34]. The SMART and SALT-ED trials demonstrated that balanced crystalloids were associated with lower rates of major adverse kidney events compared to saline in critically ill and non-critically ill adults, respectively [18,23]. However, in specific clinical scenarios such as hypochloremic metabolic alkalosis or cerebral edema, saline may remain the appropriate choice.

**Recognition of Bidirectional Relationships.** Clinicians must recognize that chloride disturbances are bidirectionally linked with organ dysfunction. Hyperchloremia may contribute to acute kidney injury through renal vasoconstriction and reduced glomerular filtration rate, while AKI itself impairs chloride excretion, perpetuating hyperchloremia [61,101]. Similarly, hypochloremia may reflect underlying severity of illness, ongoing losses, or iatrogenic causes such as diuretic therapy. Understanding these relationships enables more targeted interventions.

**Awareness of Measurement Artifacts.** Pseudohypochloremia can occur in the presence of severe hyperlipidemia or hyperproteinemia when indirect ion-selective electrode methods are used. Clinicians should be aware of this potential artifact and consider direct measurement methods or correction formulas when discrepancies between clinical presentation and laboratory values arise.

**Principles for Gradual Correction.** When correcting chloride disturbances, a gradual approach is advisable to avoid rapid shifts in acid-base status and serum osmolality. The rate of correction should be guided by the severity of the disturbance, the patient’s clinical status, and concurrent electrolyte abnormalities. There are currently no established guidelines specifying optimal correction rates for dyschloremia, representing an area requiring further research.

**Integration with Fluid Stewardship.** Chloride management should be integrated within a broader framework of fluid stewardship, as proposed by Malbrain and colleagues [34]. The four D’s of fluid therapy—drug (type of fluid), dosing (volume), duration (timing), and de-escalation (when to stop or remove fluid)—provide a conceptual structure for rational fluid prescription. The four phases of fluid therapy—resuscitation, optimization, stabilization, and evacuation (ROSE)—further guide clinicians in adapting their approach to the patient’s evolving hemodynamic status. Within this framework, chloride-conscious fluid selection becomes an integral component of high-quality critical care.

**Institutional Protocol Development.** Healthcare institutions should consider developing standardized protocols for chloride monitoring and management. Such protocols may include automatic laboratory alerts for chloride values outside the normal range, decision-support tools for fluid selection based on electrolyte status, and quality metrics to track chloride-related outcomes [106]. Integration of chloride management into existing sepsis bundles and AKI prevention strategies may enhance consistency and improve patient outcomes [34,107].

## 11. Conclusions

Chloride should no longer be considered the “forgotten electrolyte”, as it has become a clinically significant and independent prognostic biomarker in critically ill patients. The evidence synthesized in this review supports several key conclusions.

First, both hypochloremia and hyperchloremia are prevalent in the ICU setting and independently associated with increased morbidity and mortality across diverse clinical scenarios. The relationship between serum chloride and outcomes appears non-linear, with deviations in either direction conferring prognostic significance.

Second, iatrogenic hyperchloremia, primarily driven by liberal use of chloride-rich fluids, contributes to metabolic acidosis and acute kidney injury. Current evidence supports preferential use of balanced crystalloid solutions in most critically ill patients, although individualized fluid selection remains essential.

Third, serum chloride should be monitored routinely and interpreted in conjunction with sodium, acid-base status, and clinical context. Clinicians must remain aware of potential measurement artifacts that may lead to inappropriate therapeutic interventions.

Fourth, correction of chloride disturbances should be gradual, cause-directed, and integrated into comprehensive fluid and electrolyte management.

Finally, integration of chloride monitoring into institutional protocols and clinical decision support systems holds promise for improving outcomes.

Future research should focus on defining optimal chloride targets, validating chloride-guided fluid strategies, and elucidating the independent pathophysiological mechanisms underlying chloride-associated outcomes.

## Figures and Tables

**Table 1:** Classification of hypochloremia.

Severity	Chloride Values
Mild	90–95 mEq/L
Moderate	80–89 mEq/L
Severe	<80 mEq/L

**Table 2:** Classification of hyperchloremia.

Severity	Chloride Values
Mild	106–110 mEq/L
Moderate	111–115 mEq/L
Severe	>115 mEq/L

**Table 3:** Chloremia disturbances in critical care clinical scenarios.

Clinical Scenario	Main Alterations	Key Study Findings
**Sepsis**	Both hyper- and hypochloremia 12.6% with hypochloremia on admission Significant hemodynamic alterations	MIMIC-IV Study (n = 17,743): HR 0.66 for 365-day mortality in highest vs. lowest quartile Critical threshold: 105 mEq/L (L-shaped relationship) Class 4 (elevated chloride): HR 2.04 for 28-day mortality
**renal disease (AKI)**	Bidirectional relationship with AKI	471 patients on CVVH: HR 1.001 for 1-year mortality per daily chloride intake
**Surgery/Trauma**	Hyperchloremia = risk factor	Association with eGFR at 90-day follow-up
**Cardiovascular Disease**	Impaired renal excretion	10,996 patients: HR 0.65–0.71 for mortality (high vs. low quartile)
**Pulmonary Disease**	Hyperchloremic metabolic acidosis	Protective threshold: 104 mEq/L
**Cerebrovascular Disease**	Postoperative losses	Myocardial infarction: Na/Cl > 1.37 = HR 1.46 for mortality
**COVID-19**	Massive resuscitation	Heart failure: U-shaped relationship, optimal threshold 105.017 mmol/L
**Pediatric Population**	Diuretic-induced depletion	Hypochloremia: OR 1.54 for mortality

## Data Availability

No new data were created or analyzed in this study. Data sharing is not applicable to this article.
